# Low-grade fibromyxoid sarcoma of the scrotum: a rare case report

**DOI:** 10.1093/omcr/omaf234

**Published:** 2025-12-26

**Authors:** Fatima Ezzahra Laparde, Nora Naqos, Othmane Zouiten, Leila Afani, Mohamed El Fadli, Ismail Essadi, Rhizlane Belbaraka

**Affiliations:** Medical Oncology Department, Mohamed VI University Hospital of Marrakech, University Cadi Ayyad, Marrakesh, Morocco 40000, Morocco; Medical Oncology Department, Mohamed VI University Hospital of Marrakech, University Cadi Ayyad, Marrakesh, Morocco 40000, Morocco; Medical Oncology Department, Mohamed VI University Hospital of Marrakech, University Cadi Ayyad, Marrakesh, Morocco 40000, Morocco; Medical Oncology Department, Mohamed VI University Hospital of Marrakech, University Cadi Ayyad, Marrakesh, Morocco 40000, Morocco; Medical Oncology Department, Mohamed VI University Hospital of Marrakech, University Cadi Ayyad, Marrakesh, Morocco 40000, Morocco; Medical Oncology Department, Avicenna Military Hospital of Marrakech, Marrakesh 40160, Morocco; Medical Oncology Department, Mohamed VI University Hospital of Marrakech, University Cadi Ayyad, Marrakesh, Morocco 40000, Morocco

**Keywords:** scrotum, sarcoma, fibro-myxoid, soft tissue

## Abstract

Low-grade fibromyxoid sarcoma (LGFMS) is a rare soft-tissue tumor that mainly affects young adults. We present a rare case of LGFMS with scrotal involvement in a 66-years-old man. Our patient presented with a large, painless scrotal mass exhibiting pelvic extension, which was deemed inoperable. He underwent combined chemoradiotherapy. In the light of this case and a literature review, we discuss diagnostic and therapeutic approaches to establish optimal management strategies for LGFMS tumors.

## Introduction

Low-grade fibromyxoid sarcoma (LGFMS) is a rare subtype of sarcoma that mostly affects young adults [1], typically arising in the deep soft tissues of the limbs or trunk [2].

Despite its slow growth and low-grade histological appearance, LGFMS carries a significant risk of local recurrence and late metastasis. Its cause is unknown, and it accounts for only 0.6% of all sarcomas [3]. Surgical resection with clear margins is the standard treatment for localized disease. However, there is currently no evidence to support systemic or locoregional therapies for advanced or metastatic cases.

Here, we present a rare case of scrotal LGFMS.

## Case presentation

A 66-year-old bricklayer with no significant medical history presented with a painful inguinal mass persisting for 1.5 months, unresponsive to analgesics, affecting his daily activities and prompting medical consultation.

Clinical examination revealed an inguinal hernia and a right scrotal mass, which had progressively enlarged over 18 months without causing pain or functional impairment. Due to cultural and religious reasons, the patient delayed seeking medical attention.

The patient underwent laparotomy with inguinal hernia repair, but the right scrotal mass was unresectable. A biopsy confirmed low-grade fibromyxoid sarcoma, and the patient was referred to our center for further management.

**Figure 1:**
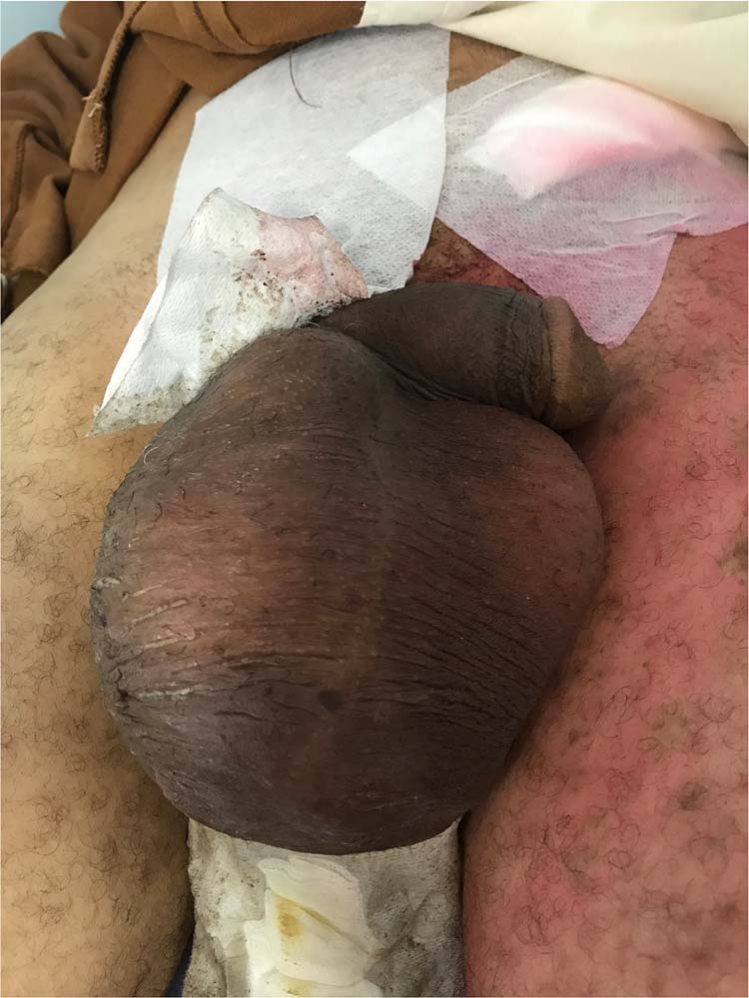
A hard, large, painless mass, lateralized to the right side of the scrotum, with the testis and penis in normal anatomical position.

On admission, the patient had a good Performance Status with stable vital signs. Examination revealed a large, hard, painless right scrotal mass (20 × 10 cm) without skin changes or lymphadenopathy. The testes and penis were normally positioned. The remainder of the examination was unremarkable except for a postoperative inguinal scar (Fig. 1).

Biopsy revealed a spindle cell proliferation within a collagenous matrix, with focal myxoid areas, cytologic atypia, and prominent vascularization. The tumor cells were arranged in haphazard, elongated fascicles and exhibited spindle-to-oval nuclei, including nuclear hyperchromasia and enlargement.

**Figure 2:**
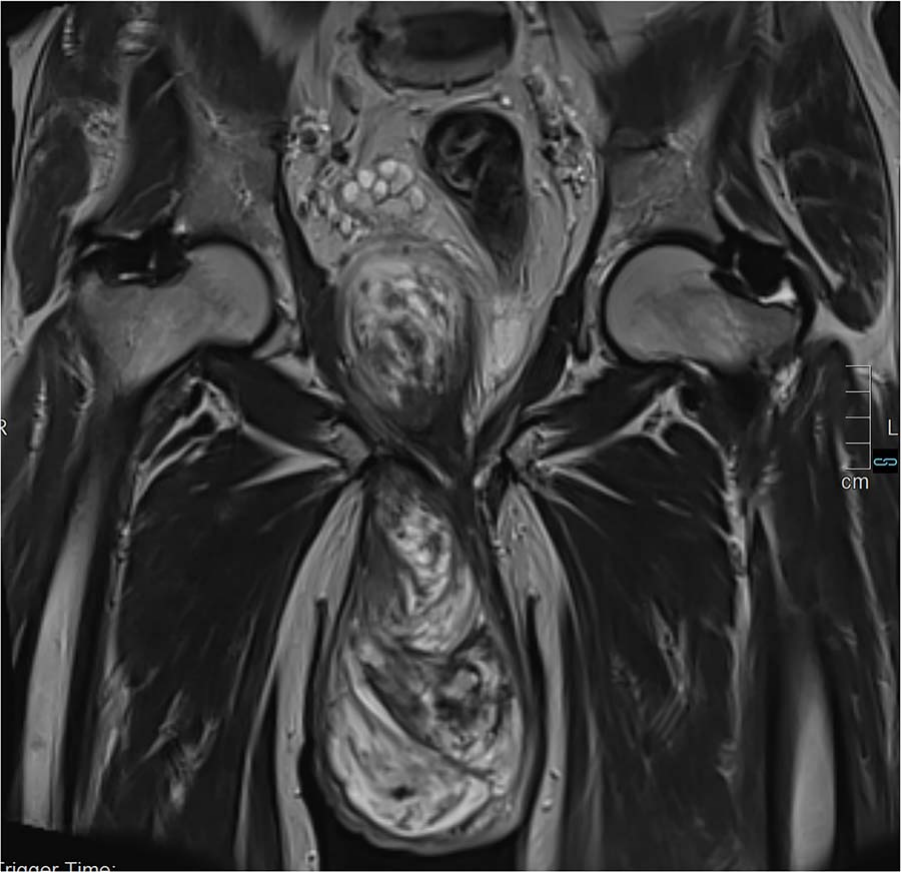
Coronal MRI section demonstrating the extent of infiltration and endopelvic extension.

**Figure 3:**
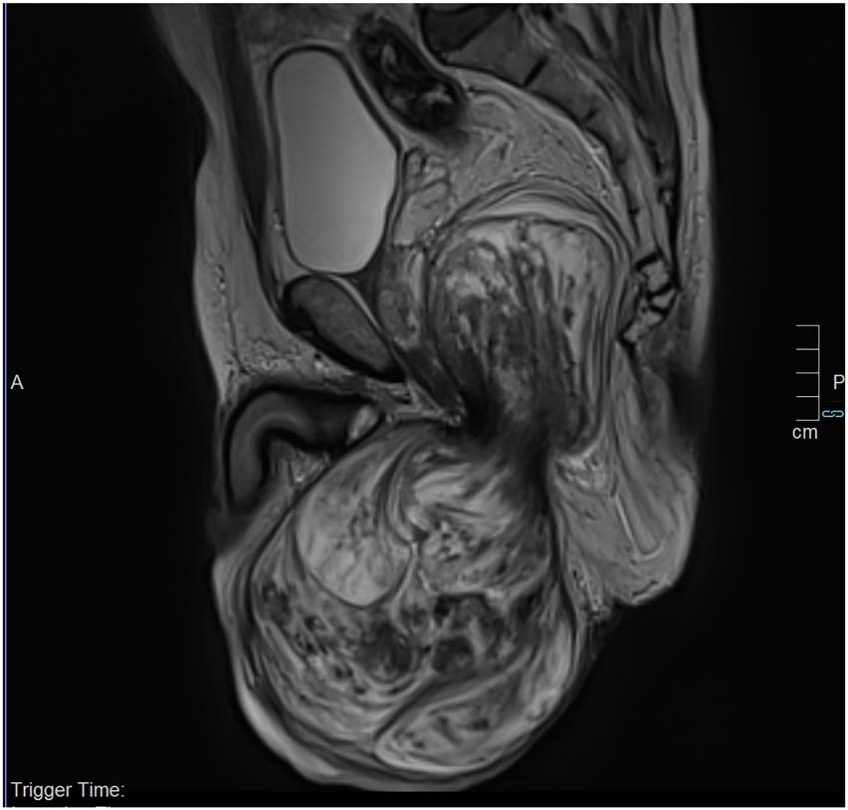
Sagittal MRI section demonstrating clear separation between the base of the penis and the bladder.

The thoraco-abdomino-pelvic CT scan showed no distant metastases. The magnetic resonance imaging (MRI) revealed a voluminous right scrotal tumor (13.5 × 9.2 × 22 cm) with endopelvic extension but no locoregional invasion (Figs. 2 and 3). The mass displaced both testicles downward while preserving the separation interface. It contacts the posterior surface of the penile base without invasion, and extends through the perineal region, occupying the right ischio-rectal fossa, displacing the rectum, prostate and seminal vesicles, but maintained clear tissue planes and distant from the bladder. Bilateral inguinal lymphadenopathy was observed, measuring 17 × 13.4 mm on the left and 20.2 × 12 mm on the right.

The patient underwent neoadjuvant chemotherapy with 4 cycles of the AIM regimen (Adriamycin 60 mg/m^2^ on Day1, Ifosfamide 1.8 g/m^2^/day from Days1–5 with Mesna), which was well tolerated both clinically and hematologically.

After four chemotherapy cycles, despite clinical findings of tumor softening and slight size reduction, imaging confirmed stable disease with persistent bilateral inguinal lymphadenopathy.

An R0 resection was not feasible due to extensive endopelvic involvement, compression of adjacent structures, and the expected negative impact on postoperative quality of life.

**Figure 4:**
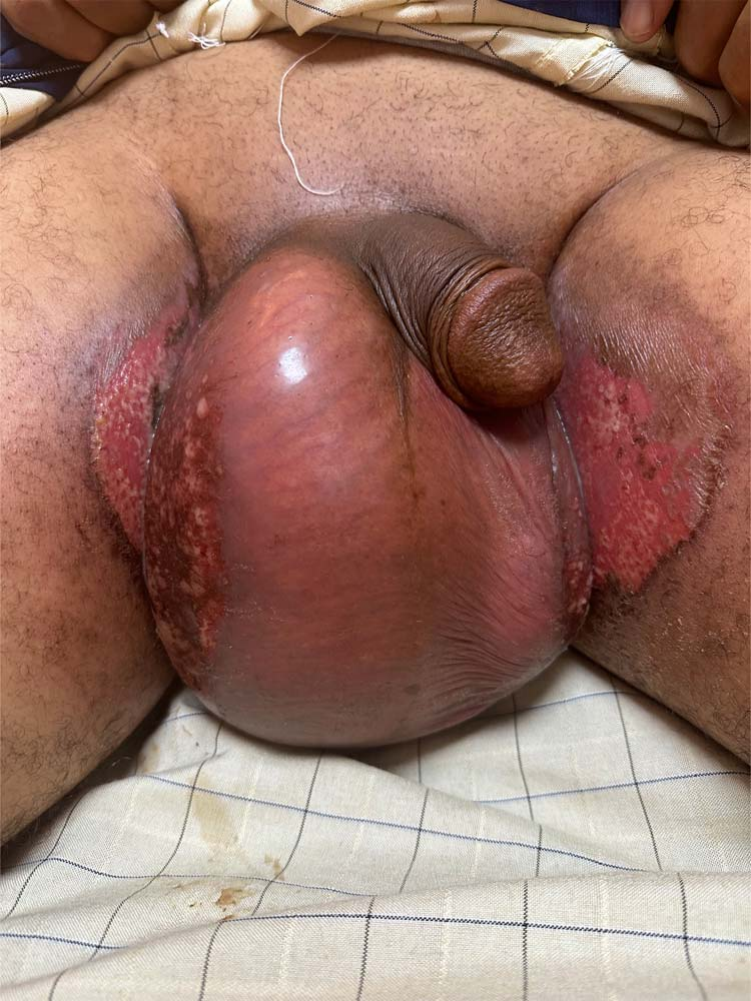
Radio-dermatitis G II.

To improve local control, the patient received adjuvant radiotherapy (66 Gy in 33 fractions). Grade II radiodermatitis developed but resolved with topical treatment and a 5-day treatment break (Fig. 4).

**Figure 5:**
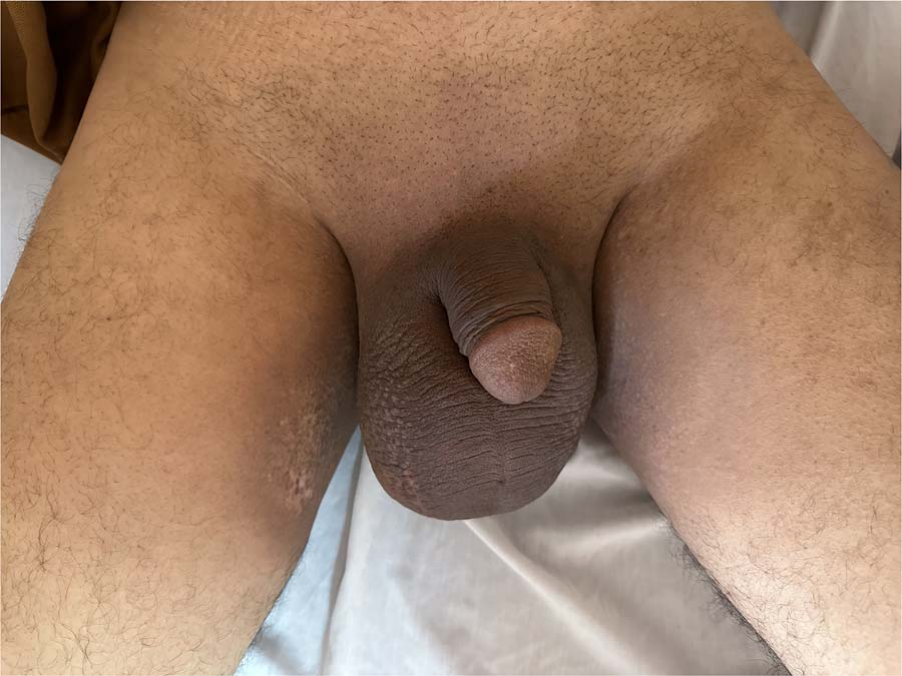
First clinical follow-up at 4 months post-radiotherapy.

The patient remains under active surveillance. At 16-month follow-up, there is no evidence of clinical or radiological disease progression (Fig. 5).

## Discussion

Low-grade fibromyxoid sarcoma (LGFMS) is an exceptionally rare soft tissue sarcoma subtype, first described by Evans in 1987 as a deceptively indolent neoplasm with a propensity for delayed local recurrence and distant metastasis [1]. It typically manifests as a large, slowly growing mass that may be either painful or painless. Scrotal localization is rare, with a limited cases reported in the medical literature.

The diagnostic protocol at the Aarhus Sarcoma Center involves clinical examination, MRI, chest imaging (X-ray/CT), and biopsy. MRI is particularly useful for identifying fibrous/myxoid tumor components, while CT is preferred for detecting metastases [3].

Histology remains the diagnostic gold standard for LGFMS, supported by immunohistochemistry to exclude similar conditions like fibrosarcoma and desmoid-type fibromatosis. MUC4 is the specific marker for LGFMS, but was unavailable in our laboratory. Instead, the patient showed CD99 positivity (90% sensitivity in LGFMS) and diffuse vimentin positivity [3]. Detection of the *FUS-CREB3L2* fusion gene from t(7;16)(q33;p11) may also aid in differential diagnosis [4].

LGFMS treatment should be individualized, but evidence remains limited. Complete surgical resection is the only consistently effective option for disease-free survival [5]. Radiotherapy is used when surgery is not possible, though the best way to combine treatment modalities is still uncertain. In the absence of strong clinical trial data, enrolling patients in clinical studies is recommended whenever possible.

Neoadjuvant therapy is typically considered for locally advanced, inoperable tumors, especially when limb preservation is challenging. In such cases, radiotherapy alone or combined with chemotherapy plays a central role in the treatment approach [6, 7]. The benefit of neoadjuvant chemotherapy alone in this setting remains uncertain.

While various local control techniques have been explored for sarcomas, no specific evidence or randomized trials currently support their use in LGFMS. Isolated limb perfusion with TNF-α and melphalan has shown promising results in locally advanced sarcomas, offering an alternative to radiotherapy by improving surgical margins and preserving limb function [8]. Interventional radiology methods like radiofrequency ablation and cryoablation have been investigated for oligometastatic disease, but outcomes in LGFMS such as RFA for lung metastases have been unfavourable [9].

In metastatic cases, a large single-center retrospective study published in 2020 demonstrated that conventional systemic therapy shows limited efficacy in advanced LGFMS (Doxorubicin, Ifosfamide, Liposomal Doxorubicin, Trabectedin) [9]. One patient received Pazopanib as fourth-line therapy, achieving disease stability as best response.

Post-treatment monitoring is crucial for early detection of recurrence, though evidence-based monitoring protocols remain poorly established [10].

In our case, the tumor was deemed inoperable due to endopelvic extension and high risk of R2 resection with potential morbidity. A combined chemotherapy and radiotherapy was chosen to enhance local control.

## Conclusion

LGFMS is a rare and clinically challenging malignancy with no standard treatment protocol. For locally advanced cases, combined chemoradiation therapy offers the best local control, while surgical resection remains a viable option if the tumor becomes operable.

## References

[1] Evans HL . Low-grade fibromyxoid sarcoma. A report of 12 cases. Am J Surg Pathol 1993;17:595–600. 10.1097/00000478-199306000-00007 8333558

[2] Mohamed M, Fisher C, Thway K. Low-grade fibromyxoid sarcoma: clinical, morphologic and genetic features. Ann Diagn Pathol 2017;28:60–7. 10.1016/j.anndiagpath.2017.04.001 28648941

[3] Maretty-Nielsen K, Baerentzen S, Keller J. et al. Low-grade Fibromyxoid sarcoma: incidence, treatment strategy of metastases, and clinical significance of the FUS gene. Sarcoma 2013;2013:256280. 23818812 10.1155/2013/256280 PMC3683502

[4] Gonzalez RS, Jerad M, MD. Gardner MD. 2002-2025, PathologyOutlines.com, Inc. Low grade fibromyxoid sarcoma. Disponible sur. https://www.pathologyoutlines.com/topic/softtissuelgfibromyxoid.html

[5] Périgny M, Dion N, Couture C. et al. Sarcome fibromyxoïde de bas grade : Une étude clinico-pathologique de 7 cas. Ann Pathol 2006;26:419–25. 10.1016/S0242-6498(06)70750-7 17255901

[6] Grobmyer SR, Maki RG, Demetri GD. et al. Neo-adjuvant chemotherapy for primary high-grade extremity soft tissue sarcoma. Ann Oncol 2004;15:1667–72. 10.1093/annonc/mdh431 15520069

[7] Kepka L, DeLaney TF, Suit HD. et al. Results of radiation therapy for unresected soft-tissue sarcomas. Int J Radiat Oncol Biol Phys 2005;63:852–9. 10.1016/j.ijrobp.2005.03.004 16199316

[8] Jakob J, Hohenberger P. Role of isolated limb perfusion with recombinant human tumor necrosis factor α and melphalan in locally advanced extremity soft tissue sarcoma. Cancer 2016;122:2624–32. 10.1002/cncr.29991 27197621

[9] Chamberlain F, Engelmann B, Al-Muderis O. et al. Low-grade Fibromyxoid sarcoma: treatment outcomes and efficacy of chemotherapy. In Vivo 2020;34:239–45. 10.21873/invivo.11766 31882484 PMC6984074

[10] Whooley BP, Mooney MM, Gibbs JF. et al. Effective follow-up strategies in soft tissue sarcoma. Semin Surg Oncol 1999;17:83–7. 10.1002/(SICI)1098-2388(199907/08)17:1<83::AID-SSU11>3.0.CO;2-W 10402642

